# Python code smells detection using conventional machine learning models

**DOI:** 10.7717/peerj-cs.1370

**Published:** 2023-05-29

**Authors:** Rana Sandouka, Hamoud Aljamaan

**Affiliations:** Information and Computer Science Department, King Fahd University of Petroleum and Minerals, Dhahran, Saudi Arabia

**Keywords:** Python, Code smell, Detection, Machine learning, Large class, Long method

## Abstract

Code smells are poor code design or implementation that affect the code maintenance process and reduce the software quality. Therefore, code smell detection is important in software building. Recent studies utilized machine learning algorithms for code smell detection. However, most of these studies focused on code smell detection using Java programming language code smell datasets. This article proposes a Python code smell dataset for Large Class and Long Method code smells. The built dataset contains 1,000 samples for each code smell, with 18 features extracted from the source code. Furthermore, we investigated the detection performance of six machine learning models as baselines in Python code smells detection. The baselines were evaluated based on Accuracy and Matthews correlation coefficient (MCC) measures. Results indicate the superiority of Random Forest ensemble in Python Large Class code smell detection by achieving the highest detection performance of 0.77 MCC rate, while decision tree was the best performing model in Python Long Method code smell detection by achieving the highest MCC Rate of 0.89.

## Introduction

During the software development process, several technical debts and design issues may occur while delivering the deadlines and meeting the new requirements. Technical debts are considered as one of the software terms, which refers to sacrificing one of the development criteria in order to achieve another one (*e.g.*, sacrificing the software quality to reach the deadline) (Zazworka et al., 2011). Code smells are considered as one of the code debts that may affect the software quality negatively. Code smells are poor code designs or implementations, such as writing complex code or implementing a very large class (Güzel & Aktas, 2016). The code smell can lay under different levels, class-level, method-level, or statement-level. However, code smells do not impact the system’s functionality but affect its quality. Therefore, detecting code smells in the early stages of designing is important to enhance the quality and performance of the code, and the whole system (Leopold, Mendling & Polyvyanyy, 2014). Code smells can be detected either in source code or system design manually or automatically. The main approaches of code smell detection are metrics-based, rule-based, and machine learning-based. The metrics-based approach evaluates the source code by defining code metrics (*e.g.*, lines of code) and a threshold value for each metric. Thus, the threshold value is the main factor that affects code smell detection accuracy. However, identifying the proper threshold is not a trivial task because there are no standard threshold values leading this approach to be unreliable (Lacerda et al., 2020; Menshawy, Yousef & Salem, 2021). In a rule-based approach, software engineer experts optimize a rule to define each code smell. Rules defining is done manually sometimes and has no standardization (Moha et al., 2009). Since these two approaches require effort from software engineers, Machine Learning (ML) algorithms were utilized recently in code smell detection to automate the detection process by building a complex mapping between the metrics and predictions. Moreover, ML algorithms have the capability to detect more complex code smells (Al-Shaaby, Aljamaan & Alshayeb, 2020). Based on the latest SLRs, ML algorithms demonstrated high code smells detection performance. However, most recent studies focus on code smell detection in Java object-oriented programs. Therefore, this area needs further studies to investigate code smell detection written in other programming languages (*e.g.*, Python) (Al-Shaaby, Aljamaan & Alshayeb, 2020; Azeem et al., 2019).

The main objective of this study is to fill the gap by creating a labeled Python code smells dataset and then utilizing conventional ML models as baselines for Python code smell detection. Our research objective will be achieved by investigating the following research questions.

• **RQ1:** How can we construct a labeled Python code smell dataset suitable for supervised learning and validate its quality?
• **RQ2:** What is the detection performance of Machine Learning models in Python code smell datasets?

This study has two main contributions. Firstly, create a Python code smell dataset for two different code smells from different code levels, which are Large Class and Long Method code smells. The dataset will be labeled into smelly and non-smelly samples with different code metrics as features. Secondly, utilize the created dataset in performing an empirical study to investigate the detection performance of baseline ML models in Python code smell detection, and they are decision trees (DT), random forest (RF), logistic regression (LR), support vector machines (SVM), multi-layer perceptron (MLP) and stochastic gradient descent (SGD).

The rest of this article is organized as follows: The section ‘Related Work’ summarizes related work on building code smell datasets and summarizes the studies that used ML in code smell detection. The section ‘Python Code Smell Dataset’ presents the process of building the dataset. The section ‘Empirical Study Design’ presents the design of our empirical study. The section ‘Results and Discussion’ presents the study results. The section ‘Threats to Validity’ presents the threats to validity. Finally, the section ‘Conclusion’ concludes the work with future work.

## Related Work

This section will demonstrate the studies that built code smell datasets to find the gap in the existing research. Then, studies that used ML models in code smell detection will be summarized to define the efficiency of ML models in this field and follow them to validate the dataset that will be built in this study.

### Dataset building

Based on the literature, some studies built code smells datasets to enrich the field and facilitate the process of code smells detection. Walter, Fontana & Ferme (2018) created a Java code smells dataset using 92 open-source projects from the Qualitas Corpus (Tempero et al., 2010). Then, they validated the created dataset, which contained 14 code smells using six different detectors. As well, Qualitas Corpus is used by Fontana et al. (2016) to identify four code smells using five automated code smells detectors. The authors used at least two detectors for each code smell. Then, the data was validated manually by three MSc students, and they found 1,160 out of 1,986 instances were classified incorrectly. Finally, they created a dataset of 140 and 280 positive and negative instances, respectively for each code smell (total of 420 instances per code smell). Later, Di Nucci et al. (2018) found limitations in this datasets and merged them to be more realistic. As a result, the author found the area of code smell detection still needs more research and more realistic datasets.

Lenarduzzi, Saarimäki & Taibi (2019) created a Technical Debt dataset that was collected from 33 Java projects using different tools. Then, it was evaluated through a set of automated quality-evaluation and code smell detection tools. The authors detected all code smells automatically without any manual validation. Sharma & Kessentini (2021) published QScored a large quality metrics and code smells dataset of 55,000 Java and 31 C# GitHub repositories containing 1.1 billion lines of codes. This dataset was developed automatically using code smells detection tools. The code quality information of QScored dataset includes several types of detected architecture smells, 20 types of design smells, and eleven types of implementation smells.

Palomba et al. (2018) developed a dataset with 243 instances of five kinds of code smells. The dataset was collected from 20 open-source Java software projects. After data collection, a systematic procedure was followed to validate the code smell dataset as follows: At the beginning, one author identified the five code smells in the projects. Then, the other author validated the identified smells by discarding any false positives. Hence, the likelihood of positive instances that are correctly classified will be increased.

Another dataset was created by Madeyski & Lewowski (2020), which is MLCQ dataset that contains 15,000 code samples of four code smells. MLCQ dataset was produced by expert software developers who analyzed industry-relevant, semi-industry relevant, and industry-irrelevant Java open-source projects.

Finally, Chen et al. (2016) implemented a tool for Python smell detection called Pysmell. They published a Python code smell dataset for several Python code smells. However, the published dataset contained labels (smelly or non-smelly) with a limited number of features. For instance, the Large Class dataset contained only the count lines of codes (CLOC) metric.

### Code smell detection using machine learning

Based on the literature, ML algorithms have been used in various code smells detection and demonstrated successful detection performance (Al-Shaaby, Aljamaan & Alshayeb, 2020). Kreimer (2005) proposed decision tree algorithm to detect the Big Class and Long Method code smells and achieved high accuracy. Then, Amorim et al. (2015) confirmed the effectiveness of the decision tree algorithm on four medium-scale open-source projects with 12 different code smells. Whereas Tian, Lo & Sun (2012) used SVM to detect God Class, Spaghetti Code Instances, Functional Decomposition, and Swiss Army Knife. Further, Arcelli Fontana et al. (2016) detected Data Class, Large Class, Feature Envy, and Long Method code smells using 16 machine learning algorithms with 1986 code smell samples. As a result, they found machine learning algorithms demonstrated high performance while the highest accuracy was obtained by J48, which is above 99%. Later, Kim (2017) detected six code smells (God Class, Feature Envy, Data Class, Lazy Class, and Parallel Inheritance) using Multilayer Perceptron (MLP) and achieved 99.25% accuracy. Khomh et al. (2011) and Vaucher et al. (2009) proposed Bayesian Belief Networks (BBN) for detecting God Class while Wang et al. (2012) proposed the same algorithms for detecting duplicated code. Yadav, Dewangan & Rao (2021) used six different machine learning algorithms (Naive Bayes, KNN, MLP, DT, RF and LR) to detect Data Class, God Class, Feature Envy, and Long Method Java code smells and they achieved high accuracy. As well, Dewangan & Rao (2022) applied different ML models for code smell detection and found that random forest model achieved the highest accuracy in feature-envy code smell detection with 99.12% accuracy.

Further, ensemble learning has been proposed in a number of code smell detection studies. Aljamaan (2021) used voting ensemble learning to investigate the performance of detecting different code smells from class-level and method-level code smells and found that voting ensemble learning achieved consistent performance among all code smells. In addition, Alazba & Aljamaan (2021) applied three different stacking ensembles learning for Java code smell detection with three different meta-classifiers (LR, SVM, and DT). As a result, constant high detection performance was obtained in both class-level and method-level detection with the stacking ensemble with LR and SVM meta-classifiers. Jain & Saha (2021) conducted several experiments to conclude that random forest and logistic regression models perform best in code smell detection. Further, stacking ensemble learning has always produced superior outcomes than using individual classifiers.

All previous studies used ML models to detect a single type of smell in the code element. However, Guggulothu & Moiz (2020) used multi-label classification algorithms to detect multiple code smells. The researchers detected two code smells which are Long Method and Feature Envy with respect to the correlation between the code smells.

From the summarized studies, it is clear most of the studies built code smell datasets for Java programming language and one study for C#. As well the ML models are used to detect code smells for the same programming languages. However, Vavrová & Zaytsev (2017) created a tool for automatic Python design defects detection, and Wang et al. (2021a) proposed a strategy for Python code smell refactoring, but the ML for code smell detection studies still focused on the other programming languages. Based on our observation of the existing gaps, more research is needed to examine the ML performance in detecting code smells for code written in other programming languages (*e.g.*, Python). Therefore, more datasets must be built for different programming languages with extracted code features to assist supervised ML models in code smell detection. Since recent studies did not focus on Python smells detection using ML models, more datasets are needed for this programming language. In this study, we will build a Python code smells dataset and utilize baseline ML models for Python code smells detection.

## Python Code Smell Dataset

Since most existing code smells datasets focus on the Java programming language, we built Python code smell datasets due to Python adoption as the popular language in Data Science and Machine Learning applications (Srinath, 2017). Fowler et al. (2002) identified and categorized a list of bad code smells. However, Python is a dynamic programming language with different code patterns than other programming languages, and it supports flexible grammatical structures. Thus, Python programming language has some unique code smells. Chen et al. (2016) presented 11 Python code smells based on the Python references (Beazley, 2009; Lutz, 2009). For instance, complex list comprehension (CLC) is a Python code smell that refers to a very complex list comprehension. List comprehension is a Python syntax to define a new list based on an existing list. On the other hand, there are Java code smells that have no potential to occur in Python. For example, the switch statement code smell in Java hence Python does not support the switch statements (Wang et al., 2021b). In addition, researchers started investigating the utilization of transfer learning (TL) in code smell detection to enhance the performance of detecting common code smells among different programming languages (Sharma et al., 2021). Using TL, we can detect code smells in different programming languages without creating a language-specific smell detection model from scratch. Therefore, it is crucial to produce new open access labeled Python code smells datasets for the same code smells in different programming languages to support this line of research. Therefore, in this study, we will build a dataset for two code smells based on the following criteria:

• Select code smells from different granularities (class-level and method-level) smells.
• Select the most investigated code smells in Java, since the existing literature mainly focuses on Java code smell datasets.

Based on the above, we selected Large Class and Long Method smells for the Python code smells dataset, since they are from different granularity levels and according to recent systematic literature reviews, they are the most investigated Java code smells (Azeem et al., 2019; Al-Shaaby, Aljamaan & Alshayeb, 2020). The definition of these code smells as follows (Alazba & Aljamaan, 2021):

• **Large Class:** is a class level code smell refers to a class that has become excessively huge and contains many lines.
• **Long Method:** is a method level code smell that refers to a long method that is hard to understand and implemented with many code lines.

**Figure 1:**
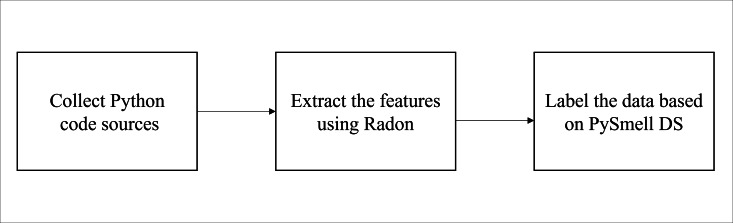
Dataset building steps.

At the beginning, Python source codes were selected and downloaded for each code smell. Then, the features were extracted for each instance of code using Radon tool (https://pypi.org/project/radon). Finally, each instance was labeled based on the published labeled PySmell dataset. The resulting dataset will contain 18 different features labeled smelly and non-smelly for each code smell. Figure 1 summarizes the followed steps for dataset building and creation. In the next subsections, we describe these steps in detail.

### Code sources selection

Our Python code smell dataset was built using four open-source Python libraries: Numpy-V1.9.2, Django-V1.8.2, Matplotlib-V1.4.3, and Scipy-V0.16.0b2. Python codes were selected based on the following criteria:

• The code must be written in Python programming language.
• The code must be open source.
• The code must be labeled (*i.e.,* smelly or non-smelly) by the PySmell dataset.

To determine the number of samples, we surveyed the most used Java code smell datasets (Fontana et al., 2016) and found their size to be 420 instances for each code smell. We choose a higher number of samples for our constructed Python code smells for better constructed supervised machine learning models. Then, we followed random sampling technique for instances selection, where we selected 1,000 samples from around 10,000 samples for the Large Class and about 40,000 samples for the Long Method. However, the number of smelly labeled instances is limited; only 200 samples were labeled as smelly for the Large Class and around 900 for the Long Method. Therefore, we limit the maximum number of smelly instances to 200. Finally, we determined the ratio to be 20% smelly and 80% non-smelly because the dataset should be imbalanced based on the code smell detection problem nature (Di Nucci et al., 2018).

### Features extraction

In order to build the Python code smell dataset, 18 different features were extracted for each code smell. The extracted features are code metrics measuring the software characteristics (*e.g.*, lines of code, number of comments, or bugs). The extracted code metrics can be classified into two types. The first type is raw metrics, which measure the general metrics that do not need complex calculations, such as the number of code lines. The second type is Halstead complexity metrics created by the late Maurice Halstead to extract a quantitative measure of complexity from the operators and operands in a code (Yu & Zhou, 2010). Table 1 presents all extracted metrics to build the dataset. Radon tool was used for metrics extraction from the source code. Radon is a well-known Python tool that analyzes and reports the codes to extract different code metrics, including: raw metrics, Cyclomatic Complexity, Halstead metrics, and maintainability metrics.

**Table 1:** The extracted code metrics for each code smell.

Metric type	Raw metrics	Halstead complexity metrics
Metrics	The number of lines of code (LOC), the number of logical lines of code (LLOC), the number of source lines of code (SLOC), the number of comment lines, the number of lines which represent multi-line strings, the number of blank lines.	The number of distinct operators, the number of distinct operands, the total number of operators, the total number of operands, program vocabulary, program length, calculated program length, volume, difficulty, effort, time required to program, number of delivered bugs.

### Dataset labeling

After collecting the Python code sources and extracting their metrics, each class and method instance was labeled smelly and non-smelly based on PySmell dataset labeling. Pysmell is a tool for Python code smell detection (Chen et al., 2016). PySmell researchers published a labeled dataset in GitHub, which was used to test their detection tool. The dataset was labeled into smelly and non-smelly for ten different Python code smells: Large Class, Long Base Class List, Long Lambda Function, Long Message Chain, Long Method, Long Parameter List, Long Scope Chain, Long Ternary, Conditional Expression, and Multiply Nested Container. Each code smell dataset contains one to three features based on the code smell (*i.e.,* code metrics). PySmell dataset was labeled by software engineer experts. While PySmell dataset has a limited number of features that could not support ML models, we combined the PySmell labels with the 18 extracted features to build a new labeled Python code smells dataset with more code features. For dataset labeling, we implemented a Python code that assigns PySmell labels to our dataset based on the library (*e.g.*, Numpy) and the method or class name.

### Dataset validation

In order to ensure the quality of the built Python code smell dataset, we considered the following criteria.

• Using a verified tool for feature extraction to ensure the quality of the code metrics. We used the Radon tool, which is an accredited Python tool.
• Using a validated and published Python code smell dataset to extract the labels. We used the Pysmell dataset, which was validated by experts.

### Dataset distribution

**Figure 2:**
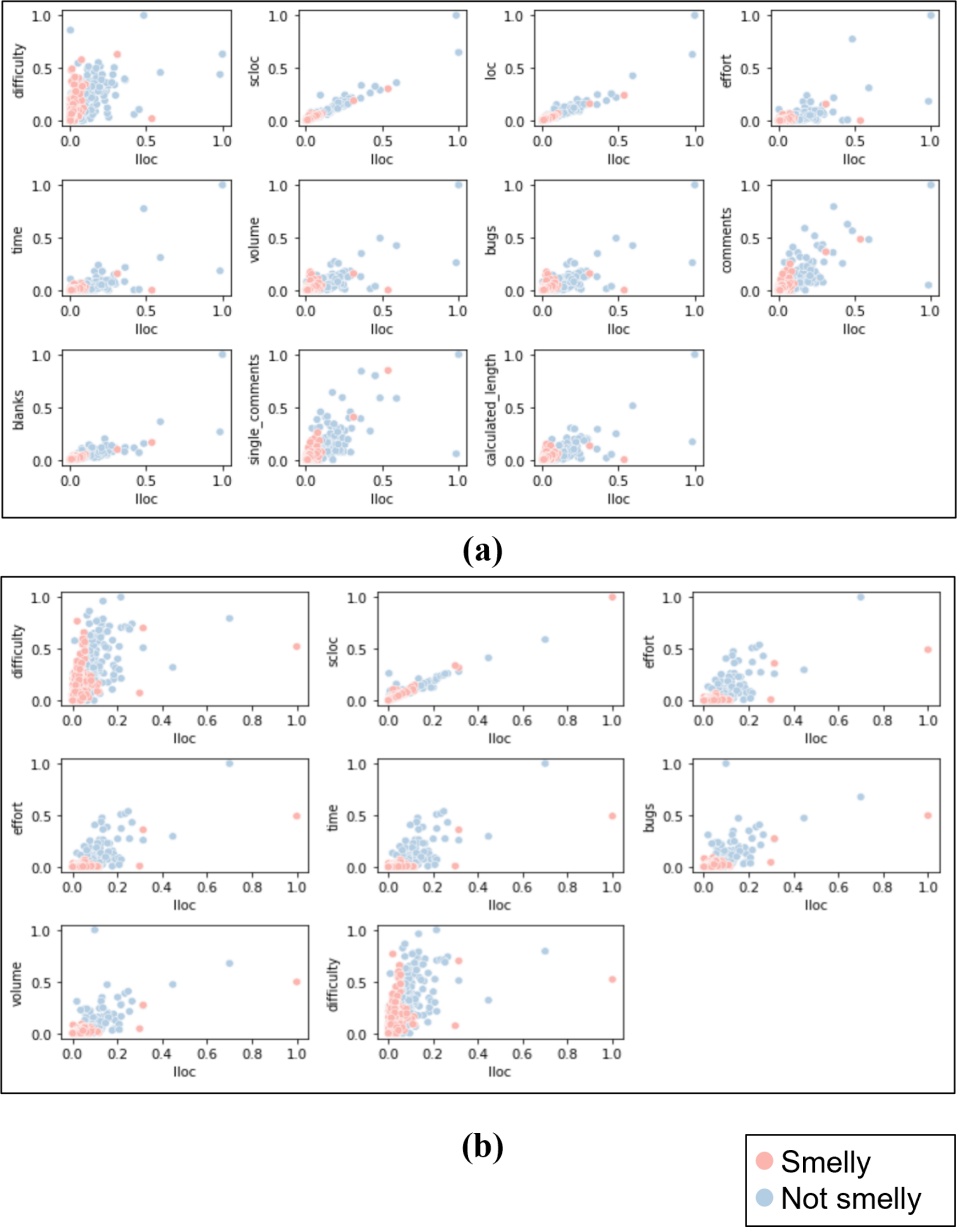
Sample of scatter plots representation for LLOC feature against the other features for both datasets. (A) Large class dataset, (B) long method dataset.

After labeling the class and method instances, a dataset of 1,000 instances was constructed for each code smell (*i.e.,* Large Class and Long Method) (https://zenodo.org/record/7512516#.ZEg3EnbMLIU). Table 2 summarizes the distribution of the Python code smells dataset. We visualized the data with scatter plots to determine whether the dataset is linear or non-linear (*i.e.,* linearly separable or not) and choose the appropriate ML models. For both datasets, each feature was plotted against the other feature with respect to the class label. In order to get better results, the data visualization was done after feature scaling and selection, which will be discussed in the next section. As a result, we found the datasets are non-linearly separable. Figure 2 shows a sample of data representation for each dataset. The figures present the LLOC feature against the other features for each dataset.

**Table 2:** The built Python dataset distribution.

Dataset	Code smell	# of features	# of smelly samples	# of non-smelly samples	Total samples
Dataset 1	Large Class	18	200	800	1,000
Dataset 2	Long Method				

BOX 1 **RQ1 Answer:** To create a Python code smells dataset, we collected open-source Python codes and extracted code metrics as features using Radon tool. Then, we utilized the labeled Pysmell published dataset instances to label our dataset. To validate the constructed dataset and ensure its quality, we labeled it based on a well-established dataset labeling, which was validated by Pysmell experts. However, we added independent features to make the dataset appropriate as input features for supervised ML models.

## Empirical Study Design

### Goal

The main goal of this study is to investigate the detection performance of ML models in detecting Python code smells using our constructed dataset. The goal is formulated using the GQM (Basili & Rombach, 1988) template as the following: ***evaluate*** machine learning models from different classification families for the ***purpose*** of Python code smells detection with ***respect*** to their detection performance measured in Accuracy and MCC from the ***perspective*** of both software engineers and researchers within the ***context*** of Large Class, and Long Method Python code smells.

### Data pre-processing

In order to enhance the ML models’ detection performance, two pre-processing steps were conducted on the collected dataset. The two steps are feature scaling and feature selection which will be explained below.

#### Feature scaling

It is a pre-processing method that aims to normalize the range of data features by making the contribution equivalent for each feature. Feature scaling is a common pre-processing practice before building machine learning models. It is known to improve data quality and ML models performance as shown in several binary classification problem studies in different domains (Chicco, Warrens & Jurman, 2021). Therefore, we applied it to improve the code smell detection performance. In this study, we followed the max-min normalization (MMN) method for feature scaling. MMN transforms the feature values into the range [0-1] where the minimum and maximum values will be 0 and 1, respectively, while the other values will be in-between. Singh & Singh (2020). The max-min normalization is presented in the following equation:

\begin{eqnarray*}{x}_{scaled}= \frac{x-min(x)}{max(x)-min(x)} . \end{eqnarray*}

#### Feature selection

It is a pre-processing method to reduce the number of features by discarding irrelevant ones and keeping only the most relevant features. Applying feature selection will increase the reliability and improve the performance of the ML models. In this study, we followed the gain ratio feature selection method. In this method, each feature will be given a gain score ranging from 0 to 1. After that, the features with a score lower than the mean gain score will be considered irrelevant and discarded from the set of features (Karegowda, Manjunath & Jayaram, 2010).

**Figure 3:**
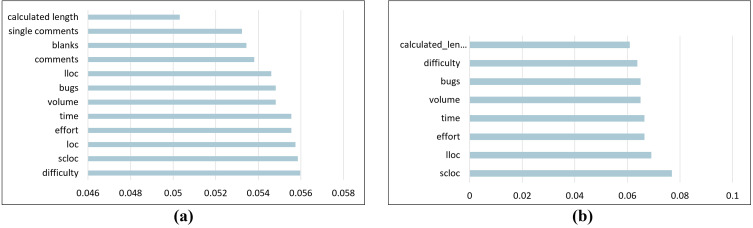
The gain ratio for each selected feature. (A) Large class dataset selected features. (B) Long method dataset selected features.

After applying feature selection, the number of features decreased based on the feature importance value, which is the gain ratio. The number of features was reduced from 18 to 12 in the Large Class dataset. The selected features are difficulty, SLOC, LOC, effort, time, volume, bugs, LLOC, number of comments, blanks, number of single comments, and calculated program length. For the Long Method dataset, the number of features was reduced from 18 to eight features: SLOC, LLOC, effort, time, bugs, volume, difficulty, and calculated program length. Figure 3 presents the gain ratio for each selected feature from highest to lowest for both Python code smells.

### Baselines

In this empirical study, we selected six ML models from different classification families as baselines to evaluate our Python code smells datasets. The used baselines are decision trees (DT), random forest (RF), logistic regression (LR), support vector machines (SVM), multi-layer perceptron (MLP), and stochastic gradient descent (SGD).

### Experiment setup

All experiments were implemented using Python because it has diverse libraries that can help with data preprocessing and developing the models. ML baseline models were built using Scikit-Learn library. It is an ML library built in Python to perform clustering, regression, and classification algorithms. Scikit-Learn has default settings of hyperparameters for each ML model. However, we fine-tuned some of them in our experiments to enhance the models’ performance. Also, Matplotlib and Seaborn libraries were used for results visualization.

### Hyperparameter optimization

ML algorithms have a set of hyperparameters that affect the algorithm’s performance. The hyperparameters of each algorithm vary based on the training dataset. In order to determine the best hyperparameters’ values, different values and combinations for each algorithm should be examined (Mhawish & Gupta, 2020). In this study, we applied the random search technique for hyperparameter optimization. Random search is one of the most popular techniques for hyperparameter optimization that involves selecting values randomly from a restricted range of hyperparameter values. The algorithm finds the hyperparameter combination that yields the highest performance after fitting the model for each combination of values using several extractions (Bergstra & Bengio, 2012). Table 3 shows the optimized hyperparameters, value ranges, and the best values selected based on the MCC metric. The tuning range was selected based on the experiments and previous work in code smell detection (Yadav, Dewangan & Rao, 2021; Yu et al., 2023).

### Model validation

ML baselines were validated using stratified 10-fold cross validation repeated ten times (10 splits and 10 repeats). In cross-validation process, the dataset is divided randomly into ten equivalent subsets. Then, nine folds were used for building the model, while the remaining fold was used for testing. Each fold is used exactly once as a testing dataset by swapping it with one of the training folds. This process was repeated ten times, and the obtained results of the iterations were averaged to get the final result. While repeated-cross-validation repeats the cross-validations process several times and addresses the performance based on the average of all folds and repeats, it can generate more accurate results and efficiently deal with overfitting with low variance to produce more reliable model performance estimates (Tantithamthavorn et al., 2016).

**Table 3:** The tuned parameters for each ML classifier.

Classifier	Hyper parameter	Default	Tuning range	Best parameter value (Large Class)	Best parameter value (Long Method)
DT	max_depth	None	(1, 100) + [None]	1	1
RF	n_estimators	100	[100, 200, 300]	200	200
LR	C	1.0	(0,1)	0.99	0.99
SVM	C	1.0	[.01, .1, 1, 5, 10, 100]	5	5
	gamma	1 / (n_features * X.var())	[0, .01, .1, 1, 5, 10, 100]	100	10
MLP	hidden_layer_sizes	100	[4, 8, 16, 32, 64, 100]	100	100

### Detection performance measures

Our study investigates Python code smells detection as a binary classification problem. ML models aim to classify code instances into smelly and non-smelly codes. In order to evaluate the models, two different evaluation measures were used: Accuracy and Matthews correlation coefficient.

#### Accuracy

It is one of the most used measures in code smell classification problems. Accuracy is the ratio of the correctly predicted samples over all data points, measured as shown in the following equation:

\begin{eqnarray*}Accuracy= \frac{TP+TN}{TP+FP+TN+FN} \times 100. \end{eqnarray*}

The higher accuracy indicates that the model achieved the highest performance in classifying the code smells into smelly and non-smelly.

#### Matthews correlation coefficient (MCC)

It is a reliable statistical measurement that produces a high score when the prediction achieves high performance in the four confusion matrix categories (true positives, false negatives, true negatives, and false positives). It returns score values between +1 and −1, where +1 indicates a perfect model, −1 indicates a perfect misclassification, and 0 indicates random prediction. Further, MCC is considered an ideal measure for imbalanced datasets because it provides high performance when the majority of each class is classified correctly. Therefore, MCC is recommended for imbalanced datasets classification more than F1-score and other measurements (Chicco & Jurman, 2020; Chicco, Warrens & Jurman, 2021). Since our Python code smell dataset is unbalanced (1/4 ratio between smelly and non-smelly), we used MCC as an evaluation metric to get accurate and stable ML model detection performance. The following equation calculates the MCC rate:

\begin{eqnarray*}MCC= \frac{TP\times TN-FP\times FN}{\sqrt{(TP+FP)(TP+FN)(TN+FP)(TN+FN)}} . \end{eqnarray*}

The higher MCC rate indicates that the model achieved the highest performance in classifying the code smells into smelly and non-smelly.

### Statistical test

To investigate the significance of detection performance differences between ML baselines, we employed the Wilcoxon signed-rank statistical test to compare the six used ML models. In order to examine if one model significantly outperforms another model, a hypothesis test is performed. The null hypothesis states, “There is no significant difference between the classifiers.”. We set the *α* value to 0.05. and examined if the *p*-value is less than 0.05, then the null hypothesis will be rejected. Otherwise, we will fail to reject the null hypothesis. After rejecting the null hypothesis, each model will be compared against the other model and the model with the highest performance will be the winner.

In this study, the models were compared against each other within each code smell dataset based on MCC scores over 100 iterations (10 Stratified CV repeated 10 times). Wilcoxon signed-rank was used since it is a non-parametric test and our results are not normally distributed (Demšar, 2006).

## Results and Discussion

Table 4 presents our empirical study results (accuracy and MCC scores) for ML baselines detection performance in detecting Python Large Class and Long Method code smells. RF model achieved the highest detection performance in Large Class code smell, while DT achieved the highest performance in Long Method code smell. In Large Class smell detection, SVM achieved the second-highest performance with marginal differences from DT, MLP, and SGD. In Long Method smell detection, RF achieved the second highest performance. On the other hand, LR had the lowest accuracy and MCC rate scores in detecting both Python code smells.

**Table 4:** Code smells detection performance results.

	**Large class**		**Long method**	
Classifier	Accuracy	MCC	Accuracy	MCC
DT	90.4	0.70	95.9	0.90
RF	92.7	0.77	95.5	0.88
LR	87.7	0.57	85.3	0.55
SVM	92.5	0.76	93.7	0.82
MLP	91.8	0.73	90.2	0.71
SGD	91.4	0.70	90.9	0.73

**Figure 4:**
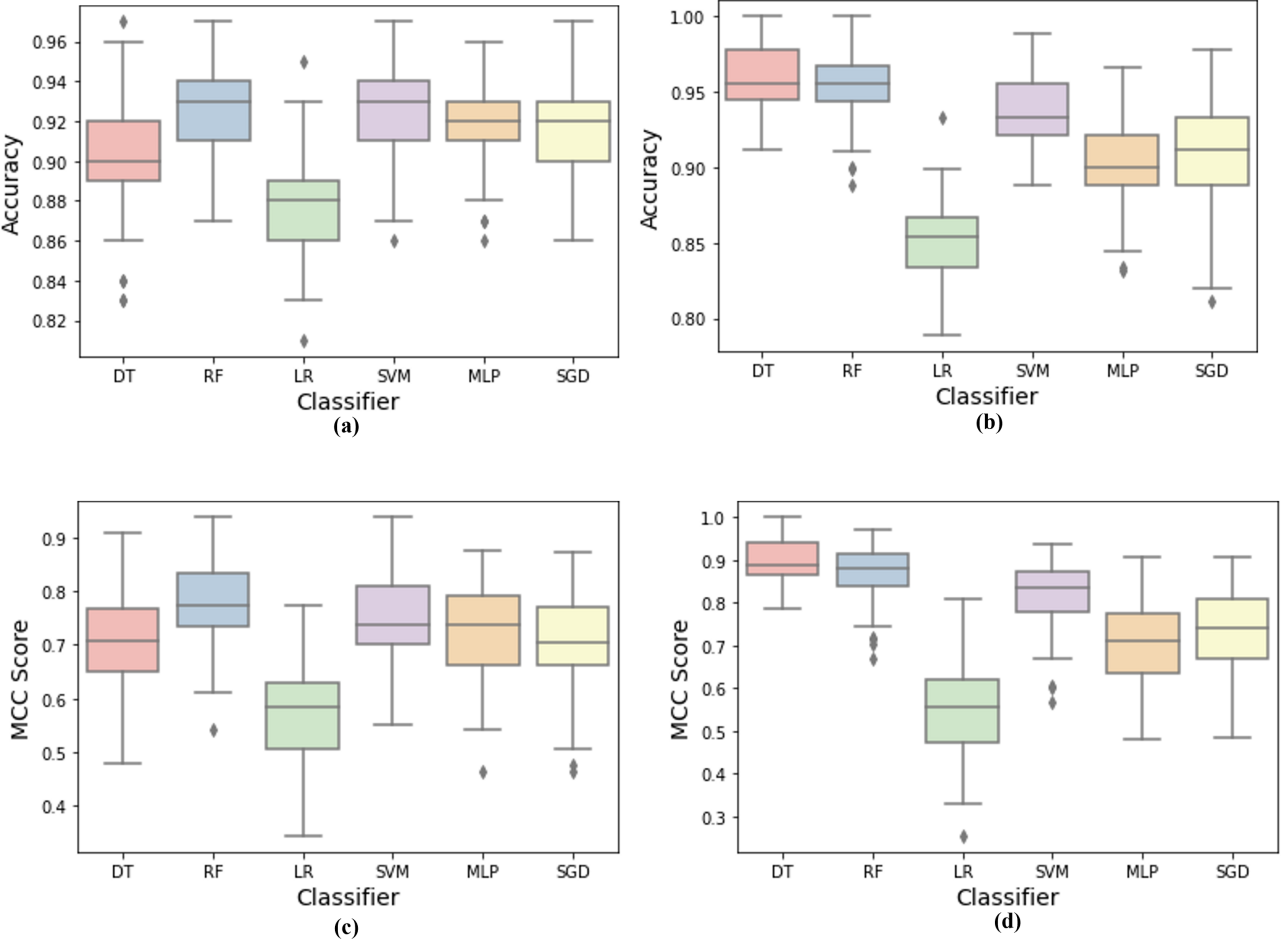
Machine learning models detection accuracy and MCC score boxplots over code smells. (A) Accuracy results for large class dataset. (B) Accuracy results for long method dataset. (C) MCC results for large class dataset, (D) MCC results for long method dataset.

Furthermore, boxplots were plotted for each code smell to examine the baseline models’ accuracy and MCC scores distribution, as shown in Fig. 4. By visually analyzing the boxplots, the models’ overall performance in detecting Long Method smell was higher than the Large Class smell. Large Class smell detection was more challenging for ML models by observing accuracy and MCC boxplots. RF model outperformed all other models in detecting Large Class code smell, while DT outperformed all other models in detecting Long Method code smell. In addition, we can observe that the RF model had more stable detection performance, as indicated by the shorter boxes and whiskers. However, it has fewer outliers in the Long Method code smell. DT and SVM models competed for the second highest MCC rate in Large Class code smell, and RF achieved the second highest performance in Long Method smell detection. On the other contrary, LR was the least performing model in terms of accuracy and MCC scores stability due to the long box and whiskers compared to other models. Finally, we can observe that most ML models’ performance in code smell detection varies from one smell type to another.

After discussing the accuracy and MCC boxplot results, we conducted the non-parametric Wilcoxon statistical test to test whether there are significant MCC rate differences between the ML models. Wilcoxon signed-rank test is a non-parametric test to compare data by analyzing the matched-pair data based on differences (Woolson, 2008). Table 5 presents the results of Wilcoxon statistical test after performing the pairwise comparison over both datasets. A pairwise comparison outcome could be a win if the first model outperforms the second model, which makes the second model a loser and the first model a winner. Outcome can be a tie if no significant difference was found between the two models (*i.e.,* fail to reject the null hypothesis) with *α* set to 0.05. To apply the pairwise comparison test, five comparisons were conducted for each machine learning model in each code smell dataset. Thus the total number of comparisons is 10. The rows of the table include numbers that represent the number of wins for each model against the other model represented in the column. In contrast, the columns of the table include the number of losses against each model represented in the row. The last two columns show the total number and the percentage of wins for each model against all other models. The last two rows show the total number and the percentage of losses for each model. As we can observe from the table, RF model won eight times against all other models (*i.e.,* 80% wins from the possible 10 comparisons). On the other hand, LR lost ten times against all other models, showing 100% losses in all pairwise comparisons over both datasets. While Wilcoxon test emphasized the difference between models’ performance based on MCC metric, we calculated the effect size to assess the difference between the models. The effect size (r) value varies from 0 to 1. Whereas 0.1 < *r* < 0.3 (small effect), 0.3 ≤ *r* < 0.5 (moderate effect) and *r* ≥ 0.5 (large effect) (Tomczak & Tomczak, 2014). Table 6 shows that RF model has large effect size against all other models for both large class and long method datasets. These results prove that the RF model outperforms the other models except DT in the Large Class dataset. On the other hand, LR was the least performing model. However, it has a small effect size against SVM and MLP in the Large Class dataset and a small effect size against SGD in the Long Method dataset.

**Table 5:** Statistical pairwise comparison results.

Classifier	DT	RF	LR	SVM	MLP	SGD	Wins	Wins%
DT &	1	2	1	1	1	6	60%
RF	1		2	1	2	2	8	80%
LR							0	0%
SVM	1		2		2	2	7	70%
MLP			2		1		3	30%
SGD			2				2	20%
Losses	2	1	10	2	6	5		
Losses%	20%	10%	100%	20%	60%	50%		

**Table 6:** Wilcoxon effect size results.

Large class						
	DT	RF	LR	SVM	MLP	SGD
DT		Small	Large	Small	Moderate	Moderate
RF	Small		Large	Large	Large	Large
LR	Large	Large		Small	Small	Large
SVM	Small	Large	Small		Large	Large
MLP	Moderate	Large	Small	Large		Large
SGD	Moderate	Large	Large	Large	Large	

Long method						
	DT	RF	LR	SVM	MLP	SGD
DT		Large	Large	Large	Large	Large
RF	Large		Large	Large	Large	Large
LR	Large	Large		Small	Small	Small
SVM	Large	Large	Small		Large	Large
MLP	Large	Large	Small	Large		Moderate
SGD	Large	Large	Small	Large	Moderate	

From the results, RF model achieved the highest performance in detecting Large Class smell and DT model was the highest achiever in Long Method smell detection based on the accuracy and MCC measures. Furthermore, the Wilcoxon test confirmed the results and showed a significant difference between the models. RF achieved the highest percentage of wins over all other models. Lastly, Large Class smell detection was more challenging to our ML baselines to achieve high detection performance compared to Long Method smell detection.

BOX 2 **RQ2 Answer:** We used a number of ML models as baselines to examine their detection performance of Python code smells. RF model was the best performing model in detecting Large Class code smells while DT model was the best performing model in detecting Long Method dataset with a marginal difference over the RF model. The second best was a competition between SVM and DT. LR was the least performing model in Python code smells detection.

## Threats to Validity

In this section, we discuss all threats to the validity of the conducted experiment including threats to external, internal, and conclusion validity.

### Internal validity

The Python code smells dataset built in this study was constructed with different code metrics as independent variables. However, there could be more effective metrics that can be utilized as features for detecting Python code smells. We selected the metrics for building the dataset based on the affordable metrics by Radon tool. Then, we refined the metrics selection using the gain ratio selection technique to validate the relationship between the independent variables (code metrics) and the dependent variable (smelly or non-smelly). We used only the metrics with a high gain ratio in our code smell detection experiments. Further, dataset labeling has been done based on PySmell dataset labels which were labeled smelly and non-smelly by experts, which could be a threat since there is no confidence degree for the labels.

### External validity

A threat to external validity is the ability to generalize our findings on the used dataset and ensure the experiment will obtain similar classification performance with different Python code smells datasets. However, we tried to mitigate this thread by collecting the dataset from four Python libraries and increasing the number of dataset instances compared to the existing Java code smells dataset.

### Conclusion validity

For conclusion threat to validity, the Python code smells dataset is considered an imbalanced dataset which could be considered as a thread that affects the experiment results. To mitigate this thread, we used Matthews correlation coefficient (MCC) metric for the models’ evaluation because it is recommended for imbalanced datasets classification. Moreover, we used Wilcoxon statistical test to examine whether the difference in detection performance between each model is significant.

## Conclusion

In this work, a labeled Python code smells dataset was built for two different code smells: Large Class and Long Method. Each dataset contains 1,000 samples labeled as 80% non-smelly and 20% smelly. Each dataset included 18 different code metrics as features. Datasets were created by collecting source code from four Python libraries: Numpy, Django, Matplotlib, and Scipy. After collecting the sources, the features extracted by Radon tool. Finally, the dataset was labeled based on the published PySmell dataset labels. After building the dataset, we preprocessed both datasets by data scaling and feature extraction. Following that, the built datasets were utilized to build ML detection models using six baseline models and evaluated their accuracy and MCC measures. Furthermore, Wilcoxon signed-rank test was conducted to examine if there are significant differences between the models or not. RF model achieved the highest performance in detecting Large Class code smell, and the DT model achieved the highest performance in detecting Long Method code smell. The achieved accuracy was 92.7% and 95.9%, while the MCC rate was 0.77 and 0.90 for Large Class using RF model and Long Method smells using DT model, respectively. Moreover, RF achieved 80% wins after applying the pairwise comparison among all models in both datasets. Lastly, we observed that detecting Large Class smell was more challenging than Long Method based on the variety of models’ performance with each code smell.

While building Python code smell datasets, we found limitations in the tools that extract the metrics of Python codes as well as tools that identify the Python code smells. These tools could help build larger datasets with more features and enhance ML models’ performance in code smells detection. As a future work, we need to build more datasets to cover more Python code smells. Furthermore, our dataset can be increased in size by increasing the number of samples and features. Finally, more ML models could be utilized to examine their performance in Python code smells detection.

##  Supplemental Information

10.7717/peerj-cs.1370/supp-1 Supplemental Information 1 Source Code

10.7717/peerj-cs.1370/supp-2 Supplemental Information 2 Wilcoxon Statistical Tests

## Additional Information and Declarations

### Data Availability

The following information was supplied regarding data availability: The data is available at Zenodo: Sandouka, Rana, & Aljamaan, Hamoud. (2023). Python Code Smell Datasets [Data set]. Zenodo. https://doi.org/10.5281/zenodo.7512516.
